# Screening of Hepatitis A and B Seropositivity among Turkish Healthcare Providers Admitted to Occupational Health Services

**DOI:** 10.1155/2022/6065335

**Published:** 2022-02-21

**Authors:** Melike Mercan Başpınar

**Affiliations:** University of Health Sciences, Gaziosmanpaşa Taksim Training and Research Hospital, Department of Family Medicine & Occupational Health and Safety Clinic, İstanbul, Turkey

## Abstract

This study aimed to determine the protection rates against hepatitis A virus (HAV) and hepatitis B virus (HBV), among healthcare providers (HCPs). The occupational health service data of Gaziosmanpaşa Training and Research Hospital between January 2020 and December 2020 were evaluated for this retrospective observational study. Of the 1,722 participants aged 34.40 ± 9.16 years, 48.6% (*n* = 861) were male, and 55.0% (*n* = 975) were doctors and nurses. The anti-HBs seropositivity rate was 87.5% (*n* = 1,501). None of the participants had anti-HCV antibodies. Twelve participants were HBsAg positive. A level of anti-HBs titer ≥10 mIU/mL was maintained in 66.7% of the HCPs vaccinated in childhood, while 71.3% (*n* = 1,263) of the participants had anti-HAV IgG. HAV vaccination needs were higher in the doctor and nurse groups than in the other groups (60.5% and 39.5%, respectively, *p* = 0.003). HBV protection was higher among HCPs in polyclinics/wards and surgery/intensive care units than in those working in the emergency department (odds ratio (OR): 2.099, 95% confidence interval (CI) = 1.285–3.429; OR: 1.592, 95% CI = 1.037–2.443, respectively). HAV protection was higher in HCPs aged 31–50 years and over 50 years than in those aged 18–30 years (OR: 2.046, 95% CI = 1.647–2.541; OR: 3.615, 95% CI = 2.164–6.037, respectively). In this study, one out of every two HCPs aged 18–30 years admitted to the occupational health control services had not yet received the HAV vaccine. The low levels of HBV protection among HCPs in the emergency department highlight the need for occupational health screening and HBV vaccination for HCPs working in emergency services in hospitals.

## 1. Introduction

The term healthcare provider (HCP) refers to all paid and unpaid people serving in healthcare settings who have the potential for direct or indirect exposure to patients or infectious materials, including body substances; contaminated medical supplies, devices, and equipment; contaminated environmental surfaces; or contaminated air [1]. Hospitals are high-risk workplace areas [2], and vaccinations for hepatitis B virus (HBV), influenza, measles, rubella, mumps, meningococcal infections, varicella, and severe acute respiratory syndrome coronavirus 2 (SARS-CoV-2) are recommended for HCPs by the Centers of Disease Control and Prevention (CDC) [1]. The national HBV vaccination program in Turkey began in July 1998 as a part of the mandatory childhood vaccine schedule [3]. The immunological memory of HBV has been reported to remain intact for at least 30 years [4], and immunocompetent individuals who achieve anti-HBs titers of ≥10 mIU/mL are protected against infection [5]. Booster doses for HCPs who initially respond to the vaccine but show a decline in antibody levels over time have been considered unnecessary by some, but several countries administer booster doses to specific risk groups [6]. The Occupational Safety and Health Administration mandates that employers offer HBV vaccination to all employees who have occupational risk and make available postexposure prophylaxis following exposure [7].

Hepatitis A is another vaccine-preventable acute infectious disease. HCPs do not have a substantially increased risk of hepatitis A virus (HAV) infection through occupational exposure, and although HAV vaccination is not routinely recommended for HCPs in the United States by the CDC [8, 9], HAV transmission from patients to HCPs is possible with fecal incontinence, inadequate hand hygiene, and sharing food or beverages [8, 10]. In 2016, the Turkish Ministry of Health requested that childhood vaccines (HAV, HBV, measles, mumps, rubella, varicella, conjugated pneumococcal, and polysaccharide meningococcal vaccines) should also be administered to eligible adults in high-risk groups, such as HCPs [11]. In addition, the HAV and HBV vaccine indications in the Turkish Viral Hepatitis Prevention and Control Program that came into force in 2018 in Turkey and the Adult Immunization Guideline 2019 have included all HCPs [3]. Therefore, occupational health screening programs for HCPs have become essential to identify individuals who require primary or booster vaccinations. Effective occupational health service programs are key to prevent exposure to infectious agents and subsequent infections [1]. Thus, employment examinations and periodic health visits for HCPs within the context of occupational health and safety screening have been implemented under the Occupational Health and Safety Law no. 6331 since 2012 in Turkey [12].

This study was performed using occupational health and safety clinical records obtained before HCPs' exposure to biological hazards as a fundamental part of the Gaziosmanpaşa Training and Research Hospital Occupational Health Surveillance Program. This study sought to evaluate HAV, HBV, and HCV seropositivity among HCPs in their first employment examination results and demonstrate the need for primary or booster vaccination in periodic health control services.

## 2. Materials and Methods

This retrospective observational study was conducted between January 01, 2020, and December 30, 2020, among 1722 HCPs who were screened for employment examinations and routine periodic occupational health visits at Gaziosmanpaşa Training and Research Hospital in Istanbul, Turkey. Exclusion criteria included the use of immunosuppressive medications, renal failure requiring dialysis, hematopoietic cell or solid organ transplantation, cancer treatment, and known immunodeficiency. Medical records were requested from the hospital, and all the available records were reviewed. One thousand seven hundred twenty-two personal records with full serology results were included in this study with the permission of hospital management. The Clinical Research Ethics Committee of Gaziosmanpaşa Training and Research Hospital approved the study protocol on 23/09/2020 (approval no. 168). The approval included a waiver of the requirement to obtain informed consent.

The outcome variables were seropositivity for HAV, HBV, and HCV, antibody statuses, and vaccine requirements. Serological markers were detected by the enzyme-linked immunosorbent assay (ELISA) (AxSYM Abbott, ARCHITECT i2000; Abbott, USA). Serum HBsAg, anti-HBs antibody, anti-HCV antibody, and anti-HAV IgG and IgM titers were recorded. Absence of HBsAg or anti-HBc and anti-HBs antibody levels of 0–10 mIU/mL for HBV, absence of anti-HCV antibodies for HCV, and absence of anti-IgG/IgM for HAV were considered to indicate seronegativity. Participants showing these findings were included in the vaccine program for the subsequent meeting during routine periodical health screening. In the control meeting of the dated sixth month, antibody titers and primary or booster vaccination status were screened again.

Normality was checked using the Kolmogorov–Smirnov test. Data were presented as mean, standard deviation, median, 25th and 75th percentiles, frequency, and percentage. Differences in variables between the two groups were analyzed using the Mann–Whitney *U* test. Variables with three or more categories were compared using Kruskal–Wallis one-way analysis of variance. Multiple comparisons were performed using Dunn's test. Nominal variables were evaluated using the chi-square and Fisher's exact probability tests. The final model for independent predictors of anti-HBs and anti-HAV seropositivity was developed using binary logistic regression analysis. Statistical significance was set at *p* < 0.05. Analyses were performed using NCSS 10 (2015, Kaysville, UT, USA).

## 3. Results

**Figure 1:**
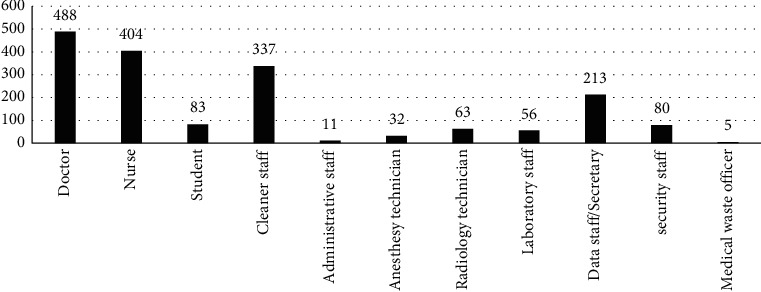
Distribution of health professions in employment examinations based on occupational health screening outcomes of the hospital in 2020.

**Figure 2:**
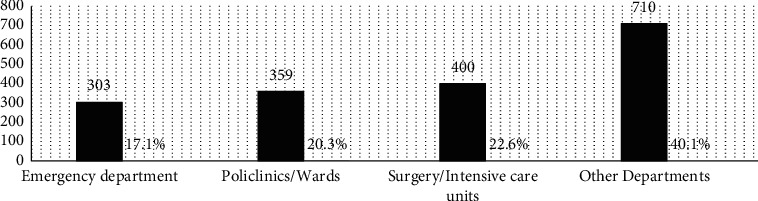
Distribution of working departments in employment examinations based on occupational health screening outcomes of the hospital in 2020.

This study included 1722 participants (age: 34.40 ± 9.16 years; male/female: 911/861), including 975 (55.0%) doctor/nurse HCPs and 797 (45.0%) other HCPs. Among the individuals, 221 (12.5%) were HBV nonimmune (anti-HBs, 0–10 mIU/mL), 420 (23.7%) were relatively immune (anti-HBs, 10–99 mIU/mL), and 1131 (63.8%) were completely immune (anti-HBs, ≥100 mIU/mL). HBsAg was detected in 12 HCPs. All HCPs showed negative results for anti-HCV antibodies. The HCPs were divided into three age groups (18–30, 31–50, and >50 years) and four primary departments (emergency, polyclinics/wards, surgery or intensive care units, and other departments). The distribution of health professions and working departments is shown in Figures 1 and 2. Among the 126 (7.1%) HCPs who received childhood HBV vaccination (born 1998 and later), 84 (66.7%) showed anti-HBs seropositivity, and 54 (42.9%) were completely immune. However, 42 (33.3%) participants were nonimmune and required booster vaccination.

Table 1 indicates that the HBV nonimmunization rate was significantly higher among males than females (55.7% and 45.3%, respectively, *p* = 0.025), and HBV vaccination needs were more frequent among the nondoctor/-nurse HCPs than the doctor/nurse HCPs (68.3% and 31.7%, respectively, *p* ≤ 0.001). The higher HBV nonimmunization rate (24%) versus protection rate (16.1%) among hospital departments was significant for the emergency department (*p* = 0.004). One out of every six HCPs (53/303) working in the emergency room was anti-HBs negative and required HBV vaccination. Analyses of the serum anti-HAV IgG antibody titers showed that 28.7% (*n* = 509) of the participants were seronegative, and 71.3% (*n* = 1263) were seropositive, although none of the participants had a history of HAV vaccination. As seen in Table 1, anti-HAV seropositivity was significantly higher in men than in women (50.8% and 49.2%, respectively, *p* = 0.003, respectively). Anti-HAV seropositivity was found more frequently in individuals aged 31–50 than in other age groups (*p* ≤ 0.001). Moreover, 54.4% of HCPs aged 18–30 years were in the anti-HAV seronegative group and were required to be included in the HAV vaccination program. HAV vaccination needs were higher in the doctor/nurse group than in the nondoctor/-nurse group (60.5% and 39.5%, respectively, *p* = 0.003).

Table 2 shows that HCPs in the emergency department had the lowest anti-HBs antibody titers (*p* = 0.008) among all departments. In addition, anti-HBs antibody titers in HCPs in the emergency department (median = 160) were lower than those in HCPs in polyclinics/wards and surgery/intensive care units (median = 215 and 295 and *p* = 0.033 and 0.001, respectively).

Table 3 shows a comparison of the serological control findings for the doctor/nurse group versus the other HCPs. The nonvaccination rate in the doctor/nurse group versus the nondoctor/-nurse HCPs was significantly more frequent for HAV vaccines (*p* ≤ 0.001) and less frequent for HBV vaccines (*p* ≤ 0.001).

Table 4 evaluates the variables of total anti-HAV seropositivity and anti-HBs seropositivity. Total anti-HBs seropositivity was found to be 2.1 times higher in polyclinics/wards, 1.6 times higher in the surgery/intensive care unit, and 1.7 times higher in other departments than in those working in the emergency department (OR: 2.099, 95% CI = 1.285–3.429; OR: 1.592 95% CI = 1.037–2.443; OR: 1.748, 95% CI = 1.194–2.558, respectively). Total anti-HAV seropositivity was 2.1 times higher in participants aged 31–50 years and 3.6 times higher in those aged over 50 years versus those aged 18–30 years (OR: 2.046, 95% CI = 1.647–2.541; OR: 3.615, 95% CI = 2.164–6.037, respectively). Total anti-HAV seropositivity was found to be 1.3 times higher in men than in women (OR: 1.277, 95% CI = 1.033–1,580). Nondoctor/-nurse HCPs had a higher anti-HAV seropositivity (OR: 1.277, 95% CI = 1.028–1.585) and a lower anti-HBs seropositivity (OR: 0.338, 95% CI = 0.249–0.461) than the doctor/nurse group.

## 4. Discussion

The occupational health and safety clinical records of 1722 HCPs at a tertiary hospital were included in this seroprevalence study. Most of the HCPs (87.5%) were anti-HBs-positive, while 63.8% were completely immune and had no need for booster HBV vaccination. Protective anti-HBs concentration (≥10 mIU/mL) was maintained in 66.7% of the HCPs vaccinated in the childhood primary vaccination schedule. Anti-HAV seropositivity was 71.3%, and HAV vaccine indication was detected in one out of every two HCPs aged 18–30 years. None of the participants showed anti-HCV positivity.

Good persistence of protective anti-HBs titers for up to 30 years with a primary vaccination without a booster dose has been demonstrated in HCPs exposed to occupational risk if their titer was initially higher than 100 mIU/mL [4]. On the contrary, according to the recommendations of the Advisory Committee on Immunization Practices (ACIP) and the European Consensus Group, HBV-vaccinated HCPs with documented immunity (anti-HBs concentrations ≥10 mIU/mL) require no postexposure prophylaxis, serological testing, or additional vaccination [5]. In a recent study at the Public Institution Health Centre of Sarajevo Canton, control measurements of protective anti-HBs titers of ≥10 mIU/mL among HCPs who received primary vaccination ten years previously showed a 74.3% seropositivity rate and a 25.7% need for booster dose [13]. While anti-HBs positivity with the vaccine was 26.4% in 1998 in Turkey, it increased to 86.9% in 2012 after the addition of the HBV vaccine to the childhood vaccination schedule [14]. The total HBV vaccination rate in this study was 12.5% (*n* = 126). While 66.7% of the HCPs who received primary childhood HBV vaccination retained HBV protection without a booster vaccine, an additional dose of the HBV vaccine was required in 33.3%. This protection rate highlights the contribution of Turkey's extended childhood vaccination program to the occupational health screening results. In a study conducted in Iran, HBV vaccine was required in 67/104 (64.4%) of the students immunized at birth [15]. This difference might be due to different types of hepatitis B vaccines in countries or other factors influencing vaccine effectiveness, such as cold-chain control systems.

In this study, the anti-HBs seropositivity rate was 87.5%, similar to the findings in recent studies conducted on HCPs in Turkey in which anti-HBs seropositivity was found in 87.5% of dental faculty students/staff [16], 89.4% of nursing students [17], and 93.7% of medical faculty students [18]. The HBV vaccination requirement rate was 12.5%. Obiri-Yeboah et al. showed an HBV vaccination requirement rate of 8.2% for HCPs in public hospitals in Ghana [19] and found differences among occupations [19]. Costa et al. observed immunity in 25.8% of the workers vaccinated for HBV and found a lower prevalence among contract workers [20]. In this study, doctors and nurses had less need for HBV vaccination than other HCPs. Additionally, the need for HBV vaccination in emergency medical services was higher than that in other departments. A systematic review suggested that emergency medical service providers are at an increased risk of contracting hepatitis B [21]. Therefore, it is not difficult to predict a high probability of transmission when dealing with critically ill patients in the resuscitation room [22]. In the present study, the number of staff with seronegativity was significantly higher than that with seropositivity. So, the emergency medical service department would benefit most from occupational disease screening in HCPs.

Grzeszczuk et al. found that the frequency of anti-HAV antibodies among Polish HCPs was 71.4%, and individuals older than 40 years were anti-HAV positive more frequently than younger individuals [23]. Erramuspe et al. found that HCPs aged under 41 years in Cordoba showed a statistically significant association with 3.57-fold higher seronegativity with a 62.2% rate of anti-HAV seropositivity [24]. HAV infection has intermediate endemicity in Turkey [25]. In a recent Turkish study, total anti-HAV seropositivity was found to be 1.73 times higher in HCPs ≥21 years old [18]. Older age has been shown to be an important factor influencing a higher rate of anti-HAV seropositivity. HCPs aged over 50 years and between 31 and 50 years had 3.6- and 2-fold higher seropositivity than those aged 18–30 years. In a study conducted with employees of a public hospital in Istanbul, anti-HAV seropositivity was significantly higher in participants aged 35 years and older than in those under 35 years of age [26]. In another study conducted with 1112 adults in Rize province, the anti-HAV seropositivity rate was 92% in people over 50 years of age [27]. In a study performed by Meryem et al., 100% anti-HAV seropositivity rate was shown in the participants aged 50 years and over, and the seropositivity rate increased with age [28]. A novel study conducted among students and staff in the dental faculty showed 31.3% anti-HAV seropositivity [16]. In different studies in Turkey, anti-HAV seropositivity among HCPs showed a broad range: 10.17% [29], 27.3% [30], 15% [31], 34.9% [18], and 64.49% [26]. In the present study, approximately three out of four HCPs (71.3%) were immune to HAV without showing an HAV vaccination history. However, one out of every two HCPs aged 18–30 years who underwent the employment examination had an HAV vaccine indication, especially for doctors or nurses among HCPs. In the future, childhood HAV vaccination may promote HAV protection in individuals aged 18–30 years, but Mosites et al. showed that the predicted seropositivity rate was 55.3% at 25 years, 49.8% at 30 years, and 45.7% at 35 years of age in a study on the immunogenicity of the hepatitis A vaccine 20 years [32]. Therefore, since the protection of childhood vaccines will decrease until the age of 30 years, it is important to determine the need for HAV vaccines in individuals aged 18–30 years.

Anti-HCV was not detected in this study population, similar to the findings of studies in Turkey [17, 31]. However, anti-HCV positivity was between 0.4% and 1.5% in community-based studies in Turkey and 1%–2.4% among HCPs [33]. Although needlesticks and contact with blood and body fluids are daily workplace risks in hospitals, the risk of HCV transmission among HCPs in Italy was found to be only 0.31% after percutaneous exposure to a hollow-bore, blood-filled needle, suggesting that HCPs may not be at high risk because of the use of needlestick prevention devices and other protective equipment [34]. Globally, injection drug use is the primary risk factor for HCV infection [35]. Therefore, we believe that the anti-HCV seronegativity in this study may be attributed to the HCV awareness of HCPs and their appropriate implementation of preventive measures, such as the use of disposable medical devices.

The main limitation of this study was the lack of data related to personal features and behavior (prior number of HBV vaccination doses for HCPs who were born before 1998, sexual behavior, hygiene habits, nicotine dependence level, alcohol consumption, and so on), which may have explained or confused the protection rates. Second, this study did not include any data to explain the high anti-HAV positivity rate without HAV vaccination, except for older age. A case-control study in India reported a significant association between HAV and a 120-fold higher risk related to a history of exposure to food from a hotel, indicating the necessity of enforcing food safety rules [36]. Thus, fecal-oral transmission sources and the factors related to these sources (geography of childhood, sanitation awareness, food habits, hygiene in hospital toilets, cafeteria, immunization of cooking chefs and food handlers at workplaces, etc.) should be investigated in a future study.

In this study, the nonvaccinated control rate in the doctor and nurse group versus other HCPs was more frequent for HAV vaccination and less frequent for HBV vaccination. Unfortunately, the retrospective study plan precluded questions about factors such as vaccine hesitancy, vaccine knowledge, and attitude.

## 5. Limitations

Although the disappearance of circulating antibodies does not necessarily mean a loss of protection, the age at which the first dose of participants was given is unclear because it was questioned whether they were only vaccinated with a full dose and what time was the last dose. Depending on mandatory childhood HBV vaccination which was introduced in Turkey in 1998, 126 participants born after this date have been considered to have received the childhood HBV vaccine in this study population including 1722 participants. So, the age distribution factor was a confounding factor in evaluating the antibody titer. In addition, since the occupational health and safety services started to function in our hospital as of 2020, the participants' protection levels and vaccination needs were scanned with the anti-HBs titer. The childhood and adolescence records of the study sample could not be standardized since the digital vaccine registration system was not widespread before 2010 when the family medicine and vaccination system was spread to all countries.

## 6. Conclusions

This study demonstrated that one out of every two HCPs aged 18–30 years who was admitted to occupational health control services required HAV vaccination, and this trend was especially prominent among doctors and nurses. The lower levels of HBV protection in HCPs working in the emergency department versus other departments highlighted the importance of occupational health screenings and HBV vaccination programs in the emergency medicine services of public hospitals.

## Figures and Tables

**Table 1:** Comparison of the preliminary screening antibody seroprotection results of health professionals on the basis of sex, age, occupation, and primary departments.

Variables	Anti-HBs antibody titer at preliminary screening (*n* = 1722)			*p* value	Anti-HAV IgG antibody titer at preliminary screening (*n* = 1722)		*p* value
		Anti-HBs seronegativity (<10 mIU/mL), *n* (%)	Anti-HBs seropositivity (≥10 mIU/mL), *n* (%)		Anti-HAV seropositivity, *n* (%)	Anti-HAV seronegativity, *n* (%)	
Sex	Male	123 (55.7%)	738 (47.6%)	*X* ^2^ = 5.048 *p* = 0.025	642 (50.8%)	219 (43.0%)	*X* ^2^ = 8.849 *p* = 0.003
	Female	98 (44.3%)	813 (52.4%)		621 (49.2%)	290 (57.0%)	

Age	18–30 years	95 (43%)	619 (39.9%)	*X* ^2^ = 3.641 *p* = 0.162	437 (34.6%)	277 (54.4%)	*X* ^2^ = 62.375 *p* ≤ 0.001
	31–50 years	117 (52.9%)	816 (52.6%)		720 (57.0%)	213 (41.8%)	
	>50 years	9 (4.1%)	116 (7.5%)		106 (8.4%)	19 (3.7%)	

Health profession area	Doctor/nurse	70 (31.7%)	905 (58.3%)	*X* ^2^ = 55.619 *p* ≤ 0.001	667 (52.8%)	308 (60.5%)	*X* ^2^ = 8.692 *p* = 0.003
	Other health professionals	151 (68.3%)	646 (41.7%)		596 (47.2%)	201 (39.5%)	

Work department in the hospital	Emergency	53 (24%)	250 (16.1%)	*X* ^2^ = 13.346 *p* = 0.004	214 (16.9%)	89 (17.5%)	*X* ^2^ = 0.758 *p* = 0.860
	Polyclinics/wards	29 (13.1%)	330 (21.3%)		262 (20.7%)	97 (19.1%)	
	Surgery units/intensive care units	50 (22.6%)	350 (22.6%)		286 (22.6%)	114 (22.4%)	
	Others	89 (40.3%)	621 (40.0%)		501 (39.75)	209 (41.1%)	

**Table 2:** Comparison of anti-HBs antibody titers (mIU/mL) according to HAV serology, health profession area, and working department in the hospital.

Variables		Anti-HBs antibody titer (mIU/ml)		
		Median	25th and 75th percentiles	*p* value
Age group	^1^18–30 years	262	49–1000	*X* ^2^ = 8.391 *p* = 0.015 ^2-1^*p* = 0.017 ^2-3^*p* = 0.033
	^2^31–50 years	179	37–816	
	^3^>50 years	255	60–1000	

Sex	Male	188	34–888	*Z* = −2.680 *p* = 0.007
	Female	256	50–1000	

Anti-HAV serology	First anti-HAV seropositivity	200	34–921	*Z* = −2.641 *p* = 0.008
	First anti-HAV seronegativity	260	64–1000	

Health profession area	Doctor/nurse	325	73–1000	*Z* = −8,050 *p* ≤ 0.001
	Nondoctor/-nurse	141	20–587	

Work department in the hospital	^1^Emergency	161	22–733	*X* ^2^ = 11.905 *p* = 0.008 ^1-2^*p* = 0.033 ^1-3^*p* = 0.001 ^3-4^*p* = 0.028
	^2^Polyclinics/wards	215	50–949	
	^3^Surgery units/intensive care units	295	50–1000	
	^4^Other departments	209	42–899	

**Table 3:** Comparison of the periodic control screening immunization results of the doctor/nurse group with those of other health professionals in the hospital.

Variables		Doctor/nurse (*n* = 975), *n* (%)	Nondoctor/-nurse (*n* = 797), *n* (%)	*p* value
HBV immunization control in periodic screening	HBV vaccinated for control screening	70 (29.9%)	164 (70.1%)	*X* ^2^ = 111.854 *p* ≤ 0.001
	HBV nonvaccinated for control screening	22 (25.9%)	63 (74.1%)	
	Protective anti-HBsAg titer	879 (61.0%)	562 (39.0%)	
	HBsAg positive members in follow	4 (33.3%)	8 (66.7%)	

HAV immunization control in periodic screening	HAV vaccinated for control screening	130 (52.6%)	117 (47.4%)	*X* ^2^ = 19.048 *p* ≤ 0.001
	HAV nonvaccinated for control screening	179 (67.3%)	87 (32.7%)	
	Protective antibody for HAV	666 (52.9%)	593 (47.1%)	

**Table 4:** Bivariate analysis of the association between participant characteristics and total anti-HAV seropositivity and anti-HBs seropositivity among healthcare providers.

Variables		Anti-HBs seropositivity Adj. OR^a^ (95% CI)	*p*	Total anti-HAV seropositivity Adj. OR^b^ (95% CI)	*p*
Sex	Female	Ref		Ref	
	Male	0.789 (0.590–1.054)	0.109	1.277 (1.033–1.580)	0.024

Age group (years)	18–30	—	—	Ref	
	31–50	—	—	2.046 (1.647–2.541)	≤0.001
	>50	—	—	3.615 (2.164–6.037)	≤0.001

Health profession	Doctor/nurse	Ref	≤0.001	Ref	
	Nondoctor/-nurse	0.338 (0.249–0.461)		1.277 (1.028–1.585)	0.027

Work department in the hospital	Emergency	Ref		—	
	Polyclinics/wards	2.099 (1.285–3.429)	0.003	—	
	Surgery units/intensive care units	1.592 (1.037–2.443)	0.033	—	
	Other departments	1.748 (1.194–2.558)	0.004	—	

^a^Adjusted according to sex, health profession, and work department in the hospital. ^b^Adjusted according to sex, age group, and health profession in the hospital.

## Data Availability

The data used to support the findings of this study are available upon request to the author.
